# Splenic injuries at Bugando Medical Centre in northwestern Tanzania: a tertiary hospital experience

**DOI:** 10.1186/1756-0500-5-59

**Published:** 2012-01-23

**Authors:** Phillipo L Chalya, Joseph B Mabula, Geofrey Giiti, Alphonce B Chandika, Ramesh M Dass, Mabula D Mchembe, Japhet M Gilyoma

**Affiliations:** 1 Department of Surgery, Catholic University of Health and Allied Sciences-Bugando, Mwanza, Tanzania; 2 Department of Surgery, Muhimbili University of Health and Allied Sciences, Dar Es Salaam, Tanzania

**Keywords:** Splenic injuries, Aetiological spectrum, Injury characteristics, Treatment outcome, Predictors of outcome, Tanzania

## Abstract

**Background:**

Splenic injuries constitute a continuing diagnostic and therapeutic challenge to the trauma or general surgeons practicing in developing countries where sophisticated imaging facilities are either not available or exorbitantly expensive. The purpose of this review was to describe our own experience in the management of the splenic injuries outlining the aetiological spectrum, injury characteristics and treatment outcome of splenic injuries in our local environment and to identify predictors of outcome among these patients.

**Methods:**

A prospective descriptive study of splenic injury patients was carried out at Bugando Medical Centre in Northwestern Tanzania between March 2009 and February 2011. Statistical data analysis was done using SPSS software version 17.0.

**Results:**

A total of 118 patients were studied. The male to female ratio was 6.4:1. Their ages ranged from 8 to 74 years with a median age of 22 years. The modal age group was 21-30 years. The majority of patients (89.8%) had blunt trauma and road traffic accidents (63.6%) were the most frequent cause of injuries. Most patients sustained grade III (39.0%) and IV (38.1%) splenic injuries. Majority of patients (86.4%) were treated operatively with splenectomy (97.1%) being the most frequently performed procedure. Postoperative complications were recorded in 30.5% of cases. The overall length of hospital stay (LOS) ranged from 1 day to 120 days with a median of 18 days. Mortality rate was 19.5%. Patients who had severe trauma (Kampala Trauma Score II ≤ 6) and those with associated injuries stayed longer in the hospital (*P *< 0.001), whereas age of the patient, associated injuries, trauma scores (KTS II), grade of splenic injuries, admission systolic blood pressure ≤ 90 mmHg, estimated blood loss > 2000 mls, HIV infection with CD4 ≤ 200 cells/μl and presence of postoperative complications were significantly associated with mortality (*P *< 0.001).

**Conclusion:**

Trauma resulting from road traffic accidents (RTAs) remains the most common cause of splenic injuries in our setting. Most of the splenic injuries were Grade III & IV and splenectomy was performed in majority of the cases. Non-operative management can be adopted in patients with blunt isolated and low grade splenic injuries but operative management is still indispensable in this part of Tanzania. Urgent preventive measures targeting at reducing the occurrence of RTAs is necessary to reduce the incidence of splenic injuries in our centre.

## Background

The spleen is the most frequently injured organ in blunt abdominal trauma, and a missed splenic injury is the most common cause of preventable death in trauma patients [1]. Despite being protected under the bony ribcage, the spleen remains amongst the vulnerable organ sustaining injury from amongst the abdominal trauma cases in all age groups [2]. It is a friable and highly vascular organ holding 25% of the body's lymphoid tissue and has both haematological and immunological functions [1-3].

Globally, splenic injuries accounts for 25% of all solid abdominal organ injuries and the mortality rate associated with splenic trauma is reported to be between 7-18% [4]. In developing countries including Tanzania, injuries in general and splenic injury in particular are increasing due to increase in urbanization, motorization, civil violence, wars and criminal activities [5]. In Bugando Medical Centre splenic injuries is the single most common cause of trauma admissions and contribute significantly to high morbidity and mortality [6].

The causes and pattern of splenic injuries have been reported in trauma literature to vary from one part of the world to another [7]. Road traffic accidents (RTAs) are the commonest cause of blunt splenic injuries in civilian practice accounting for up to 80-90% in some studies and are especially common in teenagers and young adults [7,8]. With increasing use of firearms, arrows and spears the incidence of penetrating splenic injuries has been increased in civil society [9].

In the past century, the management of splenic injury has continued to evolve from a focus almost entirely on splenectomy to one of selective non-operative management [10-13]. The risk for post-splenectomy infectious complications and the appreciation of the spleen's immunologic importance have provided an impetus to attempt spleen preservation after trauma [14]. Non-operative management of blunt splenic injuries has become the norm in Europe and North America for both children and adults because of advances in prehospital care, resuscitation, diagnostic imaging, critical care, splenorrhaphy techniques, and haemostatic agents [15]. In developing countries such as Tanzania, however, the majority of patients with splenic injuries from blunt abdominal trauma are still being managed operatively, with a low operative splenic salvage rate [5,15,16]. The lack of advanced pre-hospital and ineffective ambulance system for transportation of patients to hospital care coupled with lack of modern diagnostic imaging or inability to afford them even when available make it a great challenge to embark on non-operative management [17]. There is therefore a need to develop management protocols specific to developing countries based on categorizing the patient using clinical evaluation rather than expensive imaging if non-operative treatment is to be adopted in this region.

There is paucity of information regarding the management of splenic injuries in our environment as there is no local study which has been done in our setting particularly the study area. This study was undertaken to describe our own experiences in the management of splenic injuries, outlining the etiological spectrum, treatment outcome and prognostic factors for mortality in our local setting. The study results will provide basis for planning of prevention strategies and establishment of treatment protocols.

## Methods

### Study design and setting

This was a descriptive prospective study of patients with splenic injuries of all age groups and gender presenting to the Accident and Emergency (A&E) of Bugando Medical Centre (BMC) between March 2009 and February 2011. BMC it is located in Mwanza city along the shore of Lake Victoria in the northwestern part of Tanzania. It is a tertiary care and teaching hospital for the Catholic University of Health and Allied Sciences-Bugando (CUHAS-Bugando) and other paramedics and has a bed capacity of 1000. BMC is one of the four largest referral hospitals in the country and serves as a referral centre for tertiary specialist care for a catchment population of approximately 13 million people from Mwanza, Mara, Kagera, Shinyanga, Tabora and Kigoma.

### Patients and methods

The subjects of this study included all patients that presented to BMC with splenic injuries during the period studied and those who consented for the study. Patients who died before initial assessment and those without next of kin to consent were excluded from the study. Patients who refused to test for HIV infection were also excluded from the study. Splenic injury was diagnosed by combining clinical assessment, imaging investigations (abdominal ultrasound and Computerized tomography scan) and confirmed at surgery. Recruitment of patient to participate in the study was done at the A&E department after primary and secondary surveys done by the admitting surgical team. Patients were screened for inclusion criteria and those who met the inclusion criteria were requested to consent before being enrolled into the study. All recruited patients were first resuscitated in the A&E department according to the Advanced Trauma Life Support (ATLS) principles and were then taken into the surgical wards or the intensive care unit (ICU) from where necessary investigations were completed and further treatment was instituted. Variables studied included demographic profile (age, sex, and occupation), concomitant medical illness, mechanism and cause of injury, associated injuries, prehospital care, injury-arrival time, admission haemodynamic parameters (e.g. systolic blood pressure and pulse rate), trauma scores, Glasgow coma score, grades of splenic injury, estimated blood loss, blood transfusion requirement, treatment offered, complications of treatment. Outcome variables were length of hospital stay and mortality. The severity of injury was determined using the Kampala trauma score II (KTS II) [18]. Severe injury consisted of a KTS ≤ 6, moderate injury 7-8, and mild injury 9-10. Patients with associated head injuries were classified according to Glasgow Coma Scale (GCS) into: severe (GCS < 8), moderate (GCS 9-12) and mild (GCS 13-15). Splenic injuries were graded I to V using the Organ Injury Scaling of the American Association for the Surgery of Trauma [19]. Routine Investigations included hematological (hemoglobin, Total Leukocyte count, blood grouping & platelet count), biochemical (serum creatinine & serum electrolytes), serology (HIV testing) and radiological (X-ray chest & abdomen, abdominal ultrasound and CT scan). Depending on the grade of splenic injury, the patients were treated either non-operatively or by surgery.

Criteria for non-operative management included hemodynamic stability (defined as systolic blood pressure > 90 mmHg and pulse rate < 100 beats/min), absence of other intraabdominal injuries detected on abdominal ultrasound or CT scan requiring laparotomy, and limited need for splenic-related transfusion (≤ 2 units). Non-operative treatment was continued in patients with higher transfusion requirements only if it could be established that these additional transfusions were necessitated by associated injuries. Candidates for nonoperative management were placed on bed rest and monitored for hemodynamic stability and transfusion requirements. Patients had hourly assessment for pulse rate, blood pressure, urinary output, abdominal girth and tenderness, sensorium, temperature and respiratory rate. Daily haematocrit, blood chemistry, radiological monitor and bed rest were the routine for a period of 1 to 7 days. Patients who demonstrated any degree of hemodynamic instability or required further transfusion because of the splenic injury were immediately taken to the operating room. During surgery, the decision to perform splenectomy or to attempt splenic repair was based primarily on the grade of splenic injury, severity of associated injuries and the intraoperative stability of the patient. All patients were followed up till discharged or death. After discharge patients were followed up at the surgical outpatient clinic for up to 6 months. This information was collected using a pre-tested questionnaire.

### Statistical data analysis

Statistical data analysis was done using SPSS software version 17.0 (SPSS, Inc, Chicago, IL). Data was summarized in form of proportions and frequent tables for categorical variables. Continuous variables were summarized using means, median, mode and standard deviation. *P*-values were computed for categorical variables using Chi-square (χ^2^) test and Fisher's exact test depending on the size of the data set. Independent student t-test was used for continuous variables. Multivariate logistic regression analysis was used to determine predictor variables that are associated with outcome. A p-value of less than 0.05 was considered to constitute a statistically significant difference.

### Ethical consideration

Ethical approval to conduct the study was obtained from the CUHAS-Bugando/BMC joint institutional ethic review committee before the commencement of the study. Informed consent was sought from each patient before being enrolled into the study.

## Results

### Patient's characteristics

During the period under study, a total of 118 patients with splenic injuries were studied. One hundred and two (86.4%) patients were males and sixteen (13.6%) were females with a male to female ratio of 6.4:1. Their ages ranged from 8 to 74 years with a median age of 22 years. The modal age group was 21-30 years. Ninety-four (79.7%) were below 40 years of age, while 24 (20.3%) were aged 40 years and above

The majority of patients, 79 (66.9%) had primary or no formal education. Petty businessmen, 56 (47.5%) and students, 32 (27.1%) were commonly injured followed by peasants, pre-school children and public servants in 14(11.9%), 10(8.5%) and 6(5.1%) patients respectively. Eight (6.8%) patients reported to have concomitant illness. These included diabetes mellitus in 3 (37.5%) patients, congenital heart failure in 2 (25.0%), hypertension, renal disease and chronic chest infections in 1 (12.5%) patient each respectively. A total of 11 (9.3%) patients were HIV positive. Of these, 7 (63.6%) patients had risk factors for HIV infection such as multiple sexual partners (Odd Ratio 3.32, C.I. (2.35- 6.44), *P *= 0.002) and alcoholism (Odd Ratio 5.54, C.I. (3.67- 12.35), *P *= 0.016). Their CD4 count ranged from 78 to 696 cells/μl with the mean of 361 ± 162 cells/μl. Four (36.4%) patients in the group of HIV positive patients had CD4 count ≤ 200 cells/μL and the remaining seven (63.6%) HIV positive patients had CD4 count > 200 cells//μL.

### Circumstances of injury

The vast majority of patients, 106 (89.8%) sustained blunt injuries and the remaining 12 (10.2%) patients had penetrating injuries. The blunt to penetrating injuries ratio was 8.8:1. Road traffic accidents (RTAs) were the most common cause of injury accounting for 75 (63.6%) patients. Forty-eight (64.0%) of RTAs were related to motorcycle injuries affecting motorcyclists 22 (45.8%), passengers 16 (33.3%) and pedestrian 10 (20.8%). Other causes were fall from height, assault and sport injuries in 18 (15.3%), 12 (10.2%) and 1(0.8%) patients respectively. Penetrating injuries such as stabbing and gunshot were recorded in 12 (10.2%) patients. There were no cases of iatrogenic splenic injuries.

Most of injuries, 98 (83.1%) occurred during the day. The vast majority of patients 86 (72.9%) reported to the A & E department within 24 h after injury. The median injury-arrival time was 18 h (range: 1-268 h). None of the patients received any pre-hospital care and majority of them, 94 (79.7%) were brought in by relatives, friends or Good Samaritan, 22 (18.6%) by police and only 2 (1.7%) patients were brought in by ambulance. The median waiting time (i.e. time interval taken from reception at the A& E Department and reception of treatment) was 3 h (range 1-6 h). The majority of patients, 86 (72.9%) were attended to within 1-2 h of arrival to the A & E department

### Characteristics of the injury

Isolated splenic injuries occurred in seventy (59.3%) patients while forty-eight (40.7%) patients had multiple injuries. Associated injuries were reported in 48 (40.7%) patients the commonest being chest injuries in 75.0% of patients. The pattern of associated injuries was as shown in Table 1. Associated visceral injuries such as the liver/biliary, urinary bladder, diaphragm and the kidneys were commonly observed in blunt trauma whereas bowels and the stomach were commonly injured in penetrating trauma (Table 2).

**Table 1:** Distribution of the study subjects according to associated injuries (N = 48)

Associated injuries	Frequency	Percentage
Head	32	66.7

Chest	36	75.0

Spines	4	8.3

Pelvis	5	10.4

Musculoskeletal	28	58.3

**Table 2:** Distribution of associated visceral injuries according to the mechanism of injuries (N = 28)

Associated intra-abdominal injuries	Blunt splenic injuries (N/%)	Penetrating splenic injuries (N/%)
Liver/biliary	12 (42.9%)	11 (39.3%)

Bowels (colon/small bowel)	5 (17.9%)	7 (25.0%)

Urinary bladder	3 (10.7%)	1(3.6%)

Stomach	1(3.6%)	5(17.9%)
Diaphragm	1(3.6%)	0

Kidneys	1 (3.6%)	0

Pancreas	1 (3.6%)	1(3.6%)

According to Kampala Trauma Score II (KTS II), mild (KTS II = 9-10), moderate (KTS II = 7-8) and severe (KTS II ≤ 6) injuries were recorded in 10 (8.5%), 84(71.2%) and 24 (20.3%) patients respectively. The Glasgow coma scale in patients with head injuries indicated that most of them, 20 (62.5%) had moderate to severe injuries. The majority of patients, 92 (78.0%) had systolic blood pressure (SBP) > 90 mmHg on admission and the remaining 26 (22.0%) patients had SBP of 90 mmHg and below.

The median haemoglobin level and white blood cell count on admission were 11.2 g/dl (range 4.2-14.6 g/dl) and 12. 8 × 10^9 ^(range 3.6- 34.2 × 10^9^) respectively. The haemoglobin level was less than 10 g/dl in 62 (52.5%) patients. The total estimated blood loss in patients with splenic injuries is shown in Table 3.

**Table 3:** Estimated blood loss in patients with splenic injuries (N = 118)

Total blood loss	Frequency	Percentage
< 500 mls	10	8.5

501-1000 mls	17	14.4

1001-1500 mls	23	19.5

1501-2000 mls	42	35.5

> 2000 mls	10	8.5

Not known	16	13.6

Total	118	100

Splenic injuries were graded as follows. Four (3.4%) patients presented with Grade I, fifteen (12.7%) grade II, forty-six (39.0%) grade III, forty-five (38.1%) grade IV and five (4.2%) patients had grade V. The grade was not established in three (2.5%) patients. We observed that total number of cases in grade III and above was significantly higher than with lower grades of injuries (*P *= 0.002).

### Admission patterns and treatment parameters

The majority of patients, 97 (82.2%) were admitted in general surgical wards and the remaining 21 (17.8%) patients were admitted in the intensive care unit (ICU) where 12 (57.1%) of them were subjected to ventilatory support for a median duration of 7 days (range 2-14 days).

Of the 118 patients, 102 (86.4%) were treated operatively and the remaining 16 (13.6%) patients had non operative treatment. Of those that had operative treatment, 99 (97.1%) patients underwent splenectomy and only three (2.9%) patients had splenorrhaphy. No patient had partial splenectomy. All the patients with grade IV and V injuries had splenectomy. Of the 16 patients with non-surgically managed splenic injury, 13 (81.2%) were successfully treated without further surgical intervention. No patient in the non-operative group underwent angio-embolisation for their splenic injuries. Non-surgical management failed in 3 (18.8%) of these 16 patients 1-7 days (median, 3 days) after the initial assessment. These three patients underwent splenectomy with good results. In the group of patients who had splenorrhaphy, one patient who had grade III underwent splenectomy on postoperative day 3 due to failure of splenorrhaphy.

We noted significant increase in the rate of splenectomy during night hours (*P *= 0.023). Blood transfusion was given in 60 (58.8%) patients who were treated operatively and in only 3 (18.8%) patients who had non-operative treatment. Comparing the operative and non-operative groups, the average blood transfusion volume given were 3.0 units and 1.0 unit of packed red blood cells respectively. According to multivariate logistic regression analysis, this difference was statistically significant (*P *= 0.003). They also required a higher amount of red blood cell transfusion as compared to the non-operative group (*P *= 0.001). Operative management was more likely in patients with lower haemoglobin (*P *= 0.011) or with more severe splenic injury (*P *= 0.006). Grades I and II spleen injury was significantly associated with non-operative treatment, while grade III-V was associated with splenectomy (*P *= 0.002).

### Outcome and follow up of patients

Postoperative complications were recorded in thirty-six (30.5%) patients the commonest being surgical site infections in 38.9% of patients (Table 4).

**Table 4:** Postoperative complications among patients with splenic injuries (N = 36)

Postoperative complications	Frequency	Percentage
Surgical site infection	14	38.9

Hypovolemic shock	8	22.2

Bronchopneumonia	5	13.9

Subphrenic abscess	2	5.6

Disseminated Intravascular Coagulopathy (DIC)	2	5.6

Renal failure	2	5.6

Peritonitis	1	2.8

Intra-abdominal bleeding	1	2.8

Cardiopulmonary arrest	1	2.8

The overall length of hospital stay (LOS) ranged from 1 day to 120 days with a median of 18 days. The LOS for non-survivors ranged from 1 day to 15 days (median 5 days). The length of ICU stay ranged from 1 to 32 days (median 7 days). According to multivariate logistic regression analysis, patients who had severe trauma (Kampala Trauma Score II ≤ 6) and those with associated injuries stayed longer in the hospital and this was significant (*P *< 0.001). Comparing the non-operative and operative groups, the median length of hospital stay (17.0 versus 18.4 days) was similar (*P *= 0.005).

Of the 118, ninety-five (80.5%) patients were alive and the remaining twenty-three (19.5%) patients died. Table 5 shows predictors of mortality according to univariate and multivariate analysis.

**Table 5:** Predictors of mortality according to univariate and multivariate logistic regression analysis

Independent (predictors) variables	Survivors (N/%)	Non-survivors (n/%)	Univariate		Multivariate	
			
			O.R(95% C.I)	*P-*value	O.R(95% C.I)	*P*-value
**Age (years)**						

< 40	80 (85.1%)	14 (14.9%)				

≥ 40	15(62.5%)	9 (37.5%)	2.21(1.32-5.33)	0.032	0.22 (0.01-0.86)	**0.001**

**Sex**						

Male	82 (80.4%)	20 (19.6%)				

Female	13(81.2%)	3(18.8%)	0.23(0.11-1.52)	0.056	1.72(0.37-2.21)	0.084

**Pre-morbid illness**						

Present	6(75.0%)	2(25.0%)				

Absent	88(80.0%)	22(20.0%)	2.42 (1.95-3.34)	0.045	1.92(0.23-8.11)	0.061

**HIV status**						

Positive	6 (54.5%)	5 (45.5%)				

Negative	89(83.2%)	18(16.8%)	0.96(0.34-1.75)	0.023	1.39(1.34-1.83)	**0.012**

**CD4 count**						

≤ 200 cells/μL	1(25.0%)	3(75.0%)				

> 200 cells/μL	5(71.4%)	2(28.6%)	1.38(1.11-4.91)	0.002	2.86 (1.64-6.32)	**0.000**

**Mechanism of injury**						

Blunt	85(80.2%)	21(19.8%)				

Penetrating	10(83.3%)	2(16.7%)	1.72(0.99-2.88)	0.056	0.98(0.66-2.86)	0.067

**Injury-arrival time (hours)**						

≤ 24	69(80.2%)	17(19.8%)				

> 24	26(81.2%)	6(18.8%)	3.28(0.43-4.21)	0.054	1.93(0.98-1.64)	0.062

**Associated injuries**						

Present	32(66.7%)	16(33.3%)				

Absent	63(90.0%)	7(10.0%)	1.84(1.12-4.28)	0.011	4.81(3.88-8.55)	**0.004**

**KTSII**						

7-10	85(90.4%)	9(9.6%)				

≤ 6	10(41.7%)	14(58.3%)	2.98(1.54-5.22)	0.002	1.98(1.11-3.44)	**0.001**

**Splenic injury grade**						

I	4(100%)	-				

II	14(93.3%)	1(6.7%)	1.98(1.43-4.91)		2.92(1.11-6.32)	

III	35(76.1%)	11(23.9%)	0.23(0.12-0.83)		5.92(2.83-6.99)	

IV	38(84.4%)	7(15.6%)	1.32(1.11-2.84)		0.34(0.13-0.95)	

V	1(20.0%)	4(80.0%)	2.98(1.84-3.99)	< 0.05	0.21(0,11-0.97)	**< 0.000**

**Admission SBP**						

≤ 90 mmHg	16(61.5%)	10(38.5%)				

> 90 mmHg	79(85.8%)	13(14.1%)	1.08(1.01-3.89)	0.013	5.21(2.73-6.98)	**0.022**

**Estimated blood loss**						

≤ 2000 mls	76(82.6%)	16(17.4%)				

> 2000 mls	5(50.0%)	5(50.0%)	4.28(3,21-6.45)		1.81(1.14-6.11)	

Not estimated	14(87.5%)	2(12.5%)	6.25(3.27-8.93)	< 0.05	3.23(1.29-4.94)	**< 0.001**

**Postoperative complications**						

Present	21(58.3%)	15(41.7%)				

Absent	74(90.2%)	8(9.8%)	8.12(3.89-9.93)	0.014	3.91(1.99-5.66)	**0.010**

Key: *O.R*. = Odd ratio, *C.I*. = Confidence interval, *SBP *= Systolic blood pressure

Of the survivors, seventy-two (80.0%) patients were discharged well, fifteen (15.8%) patients were discharged against medical advice (DAMA) and the remaining four (4.2%) patients were discharged with permanent disabilities related to concomitant injuries. Out of 95 survivors, 44 (46.3%) patients were available for follow up at 3 month after discharge and the remaining 51 (53.7%) patients were lost to follow up.

## Discussion

In the present study, splenic injuries were found to be most common in the third decade of life and tended to affect more males than females. Similar demographic observation was also reported by other authors [2,7,11,13,15,16]. This group represents the economically active age and portrays an economic lost both to the family and the nation and the reason for their high incidence of splenic injuries reflects their high activity levels and participation in high-risk activities. The fact that the economically productive age-group were mostly involved demands an urgent public policy response. Male predominance in the present study is due to their increased participation in high-risk activities. Identification of risk taking behavior among trauma patients has potential significance for the prevention of injuries.

Most patients in this study sustained blunt splenic injuries, which is comparable with other studies [7,13,15] but in contrast with other studies [9,20] in which penetrating splenic injuries was the most common mechanism of injury. The high incidence of blunt splenic injuries in this study can be explained by the fact that those patients who had blunt splenic injuries were mostly involved in road traffic crash, a common feature of increased motorization in this environment. Road traffic accidents have been reported to be the commonest cause of blunt splenic injuries in most studies as supported by the present study [7,11,13,15,17]. In contrast to our findings, one study reported fall from height as the most common cause of splenic injuries [21]. High incidence of road traffic accidents in our study may be attributed to recklessness and negligence of the driver, poor maintenance of vehicles, driving under the influence of alcohol or drugs and complete disregard of traffic laws. Improvement in road conditions, prevention of overloading of commuter vehicles, maintenance of vehicles and encouraging enforcement of traffic laws will decrease the frequency and extent of these injuries.

Despite the fact that injury-arrival time did not significantly affect the outcome of our patients in term of length of hospital stay and mortality, the author of the present study still believe that prolonged injury-arrival time contributes significantly to high morbidity and mortality among patients. Early presentation to hospitals and definitive treatment of these injuries has been reported to reduce mortality and morbidity associated with the disease [7,11,13].

In the present study, none of our patients had received any pre-hospital care at the site of injury and majority of them were brought in by relatives, friends, Good Samaritan or by police who are not trained to care for trauma patients. Only 2 patients were brought in by ambulance. Similar observations have been noted in other studies in developing countries [13,15,17]. The lack of advanced pre-hospital care in our environment coupled with ineffective ambulance system for transportation of patients to hospitals are a major challenges in providing care for trauma patients and have contributed significantly to poor outcome of these patients due to delay in definitive management.

The pattern of associated injuries in this study is in agreement with findings from other studies done elsewhere [13,22]. The presence of associated injuries is an important determinant of the outcome of splenic injury patients [23]. In the present study, the presence of associated injuries was found to be significantly associated with both mortality and length of hospital stay (morbidity). Early recognition and treatment of associated injuries is important in order to reduce mortality and morbidity associated with splenic injuries.

In the present study, more than 75% of patients had grade III and above splenic injuries which is agreement with other studies in developing countries [2,11,13,15]. Carlin *et al *[23] found that the need for splenectomy was most significantly correlated with higher grades of splenic injury as supported by the present study.

The prevalence of HIV infection in the present study was 9.3% that is higher than that in the general population in Tanzania (6.5%) [24]. However, failure to detect HIV infection during window period may have underestimated the prevalence of HIV infection among these patients. The high prevalence of HIV infection in our patients may be attributed to high percentage of the risk factors for HIV infection reported in the present study population. This implies that health care workers who care for these patients are at high risk of HIV transmission due to frequent contact with body fluids starting from the Accident and Emergency department to wards and in operating theatres. Thus, all trauma health care workers in this region need to practice universal barrier precautions in order to reduce the risk of exposure to HIV infection.

In recent years the policy of spleen's conservation at operation has been established due to its important role in cellular and humoral immunity and the danger of overwhelming sepsis in asplenic patients [10-14].

The recognition that patients without a spleen have an increased risk of death from overwhelming infection, led surgeons to consider methods of splenic preservation and with the introduction of the CT scan, non-operative management became popular and then predominant [25]. Today, 90% of blunt pediatric splenic injuries and about 60-70% of adult ones are managed non-operatively in the West and other developed countries [15,17,23]. Criteria used to select patients for non-operative management of splenic injuries described in the literature include hemodynamic stability on admission, grade of splenic injury, amount of haemoperitoneum seen on CT scan, age less than 55 years, ability to elicit reliable physical signs on serial physical examination, limited blood transfusion requirements, and exclusion of other injuries that may require laparotomy [26]. However, non-operative treatment of splenic injury patients remains a challenge for Africa. It is clear that as long as non-operative management is dependent on the availability of CT scanning, it cannot be offered to most injured Africans as only a tiny minority of injured Africans have access to CT scanning. Splenorrhaphy appears a better alternative. However, its success depends on operator experience and most African surgeons are unlikely to have at their disposal the technical material, like fibrin glue or dexon mesh, which makes splenorrhaphy more successful [27,28]. In the present study, more than 80% of patients were treated operatively and the majority of patients underwent splenectomy. Similar treatment pattern was observed in other studies [2,13,17,22]. High incidence of splenectomy in our study is attributed to the large number of patients with higher grades of splenic injury and low rate of splenorrhaphy in our study may be attributed to the lack of technical material, like fibrin glue or dexon mesh, which makes splenorrhaphy more successful. Also, unlike in western countries where patients present within few hours of injury and in relatively stable clinical state [15,17,23,26], most of our patients presented to the A & E department in poor clinical state necessitating emergency laparotomy. The developing nature of our health system and haemodynamic instability of these patients on presentation makes operative management inevitable. We also noted that the time of operative intervention in our review showed an increase in the night-time splenectomy rate and the fact that most of the emergency surgery at night is performed by junior surgeons who may be unfamiliar with splenic salvage techniques may have also contributed to increased rate of splenectomy. On the other hand, in the patients with lower grades of splenic injury that had operative intervention it was due to other visceral injuries. Adequate clinical assessment, vigorous resuscitation, committed monitoring and co-operation between nursing staff and patients give good results when non-operative treatment is adopted using clinical parameters as a guide [17].

Lack of dedicated trauma centres for caring of trauma patients is a major problem in our community and the intensive care unit (ICU) at our hospital is unable to cope up with a large number of trauma patients as a result majority of patients are still admitted and managed in general surgical wards which are not well equipped in managing trauma patients. In the present study, ICU admission was influenced by injury grade, amount of haemoperitoneum, transfusion requirements, presence of coagulopathy, associated injuries or presence of co-morbidity.

The presence of complications has an impact on the final outcome of patients presenting with splenic injuries as supported by the present study. Splenic injuries are commonly associated with other injuries and these may complicate the management and affect the outcome [17]. The pattern of complications in the present study is similar to what was reported by others [13,17]. Early recognition and management of complications following splenic injury is of paramount in reducing the morbidity and mortality resulting from these injuries.

The length of hospital stay has been reported to be an important measure of morbidity among trauma patients. Prolonged hospitalization is associated with an unacceptable burden on resources for health and undermines the productive capacity of the population through time lost during hospitalization and disability [29]. The overall length of hospitalization for both survivors and non-survivors in our study were found to be higher than that reported by other authors [17,22]. This can be explained by the presence of severe trauma patients and large number of patients with associated injuries.

The overall mortality rate in this study was higher than that reported elsewhere [13,22]. Factors responsible for high mortality in our study included advanced patient's age, associated injuries, trauma scores, grade of splenic injuries, admission systolic blood pressure ≤ 90 mmHg, estimated blood loss > 2000 mls, HIV infection with CD4 ≤ 200 cells/μl and presence of postoperative complications. Addressing these factors responsible for high mortality in our patients is mandatory to be able to reduce mortality associated with these injuries.

Post-splenectomy vaccination against encapsulated organisms is highly recommended for all splenectomised patients for trauma before their discharge from hospital, with re-vaccination every 5-10 years and additional antibiotic prophylaxis to compensate for the documented occasional vaccination failure [30,31]. However, in our environment, the majority of patients post splenectomy fail to attend the follow-up clinic, making further management in those patients problematic. For these reasons, every attempt must be made for splenic salvage. This observation calls for training of junior surgical staff in methods of splenic salvage (splenorrhaphy). In the present study, none of our patient received post-splenectomy vaccination probably due to lack of availability of vaccines. This makes prevention of overwhelming post-splenectomy infection in our setting more problematic. Post-vaccination health education should therefore be given to all splenectomised patients regarding the risk, the importance of prompt diagnosis and treatment of infection, and the need for strong compliance with anti-malarial prophylaxis.

Self discharge by patient against medical advice is a recognized problem in our setting and this is rampant, especially amongst trauma patients [32]. Similarly, poor follow up visits after discharge from hospitals remain a cause for concern. These issues are often the results of poverty, long distance from the hospitals and ignorance. Delayed presentation, lack of Focused Assessment using Sonography in Trauma (FAST) and irregular availability of CT scan (due to breakdown or inability of patients to afford), unavailability of interventional radiology, inadequate ICUs, limited vaccination, discharge against medical advice, and the large number of loss to follow up were the major limitations of this study. Also, since our duration of follow up was relatively short, we could not estimate the long term outcome of both surgical and non-surgical management of splenic injuries. However, despite these limitations, the study has provided local data that can be utilized by health care providers to plan for preventive strategies as well as establishment of management guidelines for patients with traumatic splenic injuries. The challenges identified in the management of patients with splenic injuries in our setting need to be addressed, in order to deliver optimal trauma care for the victims of splenic injuries.

## Conclusion

Trauma resulting from road traffic accidents (RTAs) remains the most common cause of splenic injuries in our setting. Most of the splenic injuries were Grade III & IV and splenectomy was performed in majority of the cases (97.1%). Non-operative management can be adopted in patients with blunt isolated and low grade splenic injuries but operative management is still indispensable in this part of Tanzania. The accurate identification of a patient at high risk for poor outcome is necessary for decision making. Urgent preventive measures targeting at reducing the occurrence of RTAs is necessary to reduce the incidence of splenic injuries in our centre. A well-designed randomized clinical trial comparing the short and long term outcome of non-operative versus operative treatment in these patients is highly recommended.

## Competing interests

The authors declare that they have no competing interests.

## Authors' contributions

PLC conceived the study and participated in the literature search, writing of the manuscript, editing and submission of the article. JBM, GG, ABC, RMD and MDM participated in Study design, data analysis, manuscript writing & editing and JMG was involved in study design, data analysis, coordination and supervision of manuscript writing & editing. All the authors read and approved the final manuscript
